# Extracellular Vesicles From Gastric Cancer Cells Induce PD-L1 Expression on Neutrophils to Suppress T-Cell Immunity

**DOI:** 10.3389/fonc.2020.00629

**Published:** 2020-05-13

**Authors:** Yinghong Shi, Jiahui Zhang, Zheying Mao, Han Jiang, Wei Liu, Hui Shi, Runbi Ji, Wenrong Xu, Hui Qian, Xu Zhang

**Affiliations:** ^1^Jiangsu Key Laboratory of Medical Science and Laboratory Medicine, School of Medicine, Jiangsu University, Zhenjiang, China; ^2^Zhenjiang Key Laboratory of High Technology for Basic and Translational Research on Exosomes, Zhenjiang, China; ^3^Department of Clinical Laboratory, The Affiliated People's Hospital of Jiangsu University, Zhenjiang, China

**Keywords:** extracellular vesicles, programmed death-ligand 1 (PD-L1), neutrophils, immune suppression, gastric cancer

## Abstract

Neutrophils are prominent components of solid tumors and exhibit distinct phenotypes in different tumor milieu. We have previously shown that tumor extracellular vesicles (EVs) could induce pro-tumor activation of neutrophils; however, the role of tumor EV-elicited neutrophils in tumor immunity remains unclear. Herein, we reported that gastric cancer cell-derived EVs (GC-EVs) induced the expression of programmed death-ligand 1 (PD-L1) on neutrophils. GC-EVs transported high-mobility group box-1 (HMGB1) to activate signal transducer and activator of transcription 3 (STAT3) and upregulate PD-L1 gene expression in neutrophils. Blocking STAT3 pathway and silencing HMGB1 reversed GC-EV-induced PD-L1 expression on neutrophils. GC-EV-elicited neutrophils suppressed T cell proliferation, activation, and function *in vitro*, which could be antagonized by a specific PD-L1 antibody. Furthermore, GC tissue-derived EVs also showed similar effects. Taken together, our results indicate that EVs from the GC microenvironment induce PD-L1 expression on neutrophils to suppress T-cell immunity, which provides a new insight into the pro-tumor roles of neutrophils in GC and sheds light on the multifaceted roles of EVs in orchestrating an immunosuppressive microenvironment.

## Introduction

Gastric cancer (GC) is the fifth most common malignant tumor and the third leading cause of cancer-related death worldwide (1). Although remarkable achievements in surgical and other therapies have been obtained, the prognosis of GC is still poor. In recent years, immunotherapy has emerged as an attractive therapeutic approach for cancer. Preclinical and clinical studies demonstrate that programmed death-ligand 1 (PD-L1)/programmed death-1 (PD-1) antibodies have promising anti-tumor activities in patients with cancer, including GC (2, 3). However, the resistance to PD-1/PD-L1 antibody therapy has also been observed.

PD-L1 is overexpressed in many cancers (4, 5). Tumor cells evade immune surveillance by upregulating the surface expression of PD-L1, which interacts with PD-1 on T cells to elicit the immune checkpoint response (6). Anti-PD-1 antibodies have shown remarkable effects on tumor therapy (7). However, the patient response rate is various in different cancers (8). Recently, Haderk et al. (9) demonstrate that PD-L1 is highly expressed on macrophages/monocytes, which may reveal a possible reason for the resistance to PD-L1 blockade in cancer. Thus, a better understanding of PD-L1 expression and regulation is needed to predict patient response and improve treatment efficacy.

Neutrophils are important immune cells in tumor niche and have both pro- and anti-tumor functions (10). In response to tumor signals, neutrophils are educated to facilitate tumor progression through their ability to promote tumor growth, angiogenesis, invasion, and metastasis (11). Neutrophils have also been proposed to exert pro-tumor activities by acting as immunosuppressive effectors in both innate and adaptive immune response (12). The previous study has shown that neutrophils could recruit regulatory T cells to tumor sites *via* secretion of CCL17 to impair antitumor immunity (13). Recently, neutrophils have been reported to suppress intraluminal NK cell-mediated tumor cell clearance (14). Thus, further study of the function of neutrophil in tumor immunity will provide new approaches for GC therapy.

Extracellular vesicles (EVs) are small lipid bilayer membrane vesicles and considered as an important mechanism for cellular communication, allowing cells to exchange genetic materials and signal molecules. EVs are involved in multiple physiological and pathological processes (15). Increasing evidence suggest that tumor-derived EVs reshape immune cells to help escape immune surveillance (16, 17). We have previously shown that GC cell-derived EVs could induce neutrophils N2 polarization, which in turn promotes tumor cell proliferation, migration, and invasion (18, 19). However, the function of tumor EV-elicited neutrophils in tumor immunity has not been well-characterized.

In this study, we reported that tumor EVs could induce PD-L1 expression on neutrophils. Tumor EV-delivered high-mobility group box-1 (HMGB1) activated signal transducer and activator of transcription (STAT)3 pathway in neutrophils to upregulate PD-L1 gene expression. Tumor EV-elicited neutrophils suppressed the proliferation, activation, and function of T cells in a PD-L1-dependent manner. These findings suggest that tumor EVs could reeducate neutrophils to create an immunosuppressive microenvironment for GC progression.

## Materials and Methods

### Patients and Specimens

Fresh gastric tumor and non-tumor (at least 5 cm away from the tumor site) tissues were obtained from patients with GC who underwent surgical resection at the Affiliated People's Hospital of Jiangsu University. None of these patients had received chemotherapy or radiotherapy before surgery. Patients with infectious diseases, autoimmune disease, or multi-primary cancers were excluded. The study was approved by the ethics committee of Jiangsu University. Written informed consent was obtained from all patients.

### Cell Culture and Preparation of Conditioned Medium

Human GC cell line BGC-823 was purchased from the Institute of Biochemistry and Cell Biology at the Chinese Academy of Sciences (Shanghai, China). Cells were cultured in RPMI-1640 medium, supplemented with 10% fetal bovine serum (FBS; Invitrogen, Carlsbad, CA, USA) at 37°C in humidified air with 5% CO_2_. When cells reached 80% confluence, they were changed to exosome-depleted medium and cultured for another 24 h to obtain conditioned medium (BGC-CM). Tumor tissue conditioned supernatants (TTCS) and non-tumor tissue conditioned supernatants (NTCS) were prepared by plating tumor or non-tumor gastric tissues in 1 ml exosome-depleted RPMI-1640 medium. Following a 24-h incubation, all media were collected, respectively, centrifuged to remove cell debris, and stored at −80°C in aliquots.

### Extracellular Vesicle Isolation

EVs from cultures supernatants were isolated as previously described (18). In brief, cells were cultured in exosome-depleted medium, and the supernatants were collected after 48 h. The conditioned medium was centrifuged at 1,000 g for 10 min to remove cell debris followed by 30 min at 10,000 g using 100 KDa MWCO before the concentrated solutions were filtrated through a 0.22-μm pore filter (Millipore, Shanghai, China). EVs were precipitated by adding the exosome quick extraction solution (System Biosciences, Palo Alto, CA, USA) at a ratio of 1:5 at 4°C for 16 h. For isolating EVs from tumor tissue culture supernatants, tissues were cultured in exosome-depleted medium and the supernatants were collected after 48 h followed by centrifugation at 1,000 g for 10 min to remove cell debris. EVs were precipitated by adding the serum exosome quick extraction solution (System Biosciences, Palo Alto, CA, USA) at a ratio of 1:5 at 4°C for 12 h. EVs were dissolved with phosphate buffered saline (PBS) and stored at −80°C. The isolated EVs were characterized as previously described.

### Isolation of Neutrophils and T Cells

Neutrophils were isolated by using Polymorphprep (Axis-Shield PoC AS, Norway) as previously described (19). RBCs were lysed using hypotonic lysing procedure. Peripheral blood mononuclear cells (PBMCs) from healthy volunteers were isolated by density gradient centrifugation using Ficoll-Paque gradient method. CD3^+^ T cells from PBMCs were purified by using anti-CD3 (Miltenyi Biotechnology) magnetic beads. The sorted T cells and neutrophils with a purity of higher than 95% were used.

### Treatment of Neutrophils

Neutrophils were stimulated with 200 μg EVs from supernatants of GC cells or tissues for 12 h. After stimulation, the cells were harvested for flow cytometric analysis, real-time quantitative PCR, and Western blot. For the signaling pathway inhibition experiments, neutrophils were pretreated with Janus kinase (JAK)/STAT3 inhibitor WP1066 (10 μM, Beyotime Biotechnology, Shanghai, China) for 1 h, then the cells were stimulated with EVs for 12 h and harvested as above. Neutrophils for repeated experiments were from different donors.

### Coculture of Neutrophils With T Cells

Before coculture with T cells, neutrophils were washed three times with PBS. In a 3-day incubation, bead-purified peripheral CD3^+^ T cells (1 × 10^5^ cells/well in 96-well plates) were labeled with carboxy fluorescein succinimidyl ester (CFSE, Stem Cell Technology) and cocultured with neutrophils at a 1:1 ratio in 100 μl RPMI-1640 medium containing anti-CD3 (8 μg/ml) and anti-CD28 (5 μg/ml) antibodies (eBioscience). In a 1-day coculture system, neutrophils were cocultured with bead-purified CD3^+^ T cells at a 1:1 ratio in 100 μl RPMI-1640 medium containing anti-CD3 (5 μg/ml) and anti-CD28 (2 μg/ml) antibodies (eBioscience). After 1-day incubation, the cells were harvested for flow cytometric analysis of CD69 and interferon (IFN)-γ. For blockade study, the treated neutrophils were cocultured with bead-purified CD3^+^ T cells in the presence of a neutralizing antibody against human PD-L1 (2 μg/ml) (R&D Systems).

### RNA Inference

The specific siRNAs against HMGB1 were produced by GenePharma (Suzhou, Jiangsu, China). The sequence of HMGB1 siRNA was 5′-UUCUCCGAACGUGUCACGUAA-3′, and the scramble control was 5′-CCGUUAUGAAAGAGAAAUGAAU-3′. Cells (1 × 10^6^ cells/well) were grown in six-well plates and transfected with siRNAs by using LipoFiter transfection reagent (Hanbio, Shanghai, China) for 6 h. Following a 24-h incubation, the transfected cells were harvested and replated in 10-cm plates. The conditioned media were collected as for EV isolation.

### Flow Cytometry

Flow cytometric analysis was performed according to standard protocols on multicolor flow cytometry (FACS Canto II, BD Biosciences). For analysis of immunophenotype, neutrophils were stained with phycoerythrin (PE)-conjugated PD-L1 antibodies (BioLegend), and T cells were stained with fluorescein isothiocyanate (FITC)-conjugated CD3 antibodies (BioLegend) and allophycocyanin (APC)-conjugated CD69 antibodies (BioLegend). For intracellular cytokine measurement, T cells were stimulated for 5 h with Cell Stimulation Cocktail (eBioscience). Intracellular cytokine staining was performed after fixation and permeabilization using Perm/Wash solution (FMS, China). The results were analyzed using FlowJo 10.0 software (TreeStar).

### Real-Time Quantitative PCR

Total RNA was extracted from cells using Trizol reagent (Thermo Fisher Scientific), and 1 μg of RNA was reverse transcribed to cDNA by using reverse transcriptase (Vazyme). Real-time quantitative PCR was performed by using the SYBR Green I real-time detection kit (Cwbio, Beijing, China) on a Bio-Rad CFX96 Detection System. The relative gene expression was normalized to β-actin. PCR primers were as follows: PD-L1 sense, 5′-TGGCATTTGCTGAACGCATTT-3′, and PD-L1 antisense, 5′–TGCAGCCAGGTCTAATTGTTTT−3′; β-actin sense, 5′-CACGAAACTACCTTCAACTCC-3′, and β-actin antisense, 5′-CATACTCCTGCTTGCTGATC-3′.

### Western Blot

Cells were lysed in RIPA buffer containing proteinase inhibitors. Equal amount of proteins was separated by a 10% sodium dodecyl sulfate–polyacrylamide gel electrophoresis (SDS-PAGE) gel. Following electrophoresis, proteins were transferred to a polyvinylidene fluoride (PVDF) membrane, blocked in 5% non-fat milk, and incubated with primary antibodies at 4°C overnight. Antibodies against PD-L1, STAT3, p-STAT3, and HMGB1 were purchased from Cell Signaling Technology (Louis Park, MN, USA). After washing with Tris-buffered saline + Tween 20 (TBS/T) three times, membrane was incubated with horseradish peroxidase (HRP)-conjugated goat anti-rabbit or anti-mouse secondary antibodies (Bioworld Technology) at room temperature for 2 h. The protein bands were visualized by enhanced chemiluminescence. Glyceraldehyde 3-phosphate dehydrogenase (GAPDH) served as the loading control.

### Statistical Analysis

Data were expressed as mean ± SD. The statistical significance of differences between two groups was determined by two-tailed Student's *t*-test. The significance of differences among multiple groups was determined by one-way ANOVA. All experiments were performed at least in triplicate (*n* = 3). *P* < 0.05 was considered statistically significant. All statistical analyses were calculated using GraphPad Prism (GraphPad Software, La Jolla, CA).

## Results

### Gastric Cancer-Derived Extracellular Vesicles Induce Programmed Death-Ligand 1 Expression on Neutrophils

Neutrophils are important players in tumor development and progression and could be polarized to an N2 phenotype by tumor signals (11, 20). To evaluate the potential role of human GC microenvironment on neutrophils, we verified that GC cell-derived conditioned medium (GC-CM) induced PD-L1 expression on neutrophils as reported (21) (Figure S1). However, tumor cells can release various factors to mediate cellular communication in tumor micorenvironment, including EVs (22). EVs carry bioactive molecules that can reshape tumor microenvironment (16, 23). In our previous study, we purified EVs from GC cell-derived conditioned medium and verified them by transmission electron microscopy (TEM) and nanoparticle tracking analysis (NTA). We found that GC cell-derived EVs displayed sphere-like morphology with a diameter ~100 nm and expressed the exosomal markers CD9 and CD63 (18).

**Figure 1:**
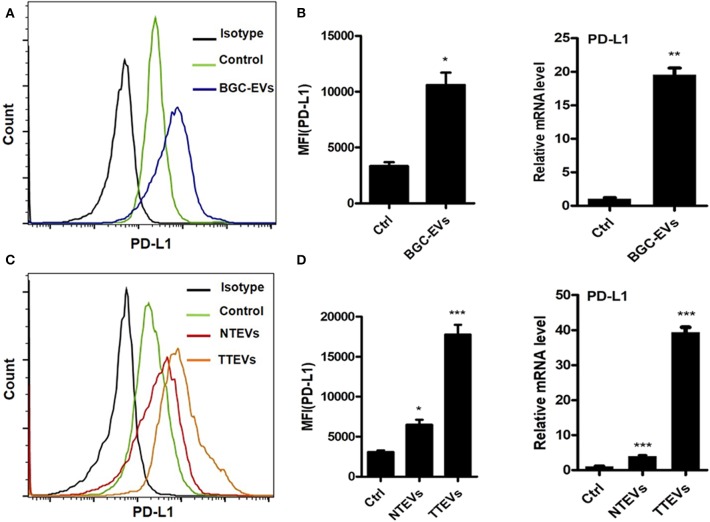
Gastric cancer (GC)-derived extracellular vesicles (EVs) induce programmed death-ligand 1 (PD-L1) expression on neutrophils. Protein and gene levels of PD-L1 on neutrophils exposed to BGC-EVs for 12 h were determined by flow cytometry **(A)** and qRT-PCR **(B)**. Flow cytometric **(C)** and qRT-PCR analyses **(D)** of protein and gene levels of PD-L1 on neutrophils exposed to NTEVs and TTEVs for 12 h. Ctrl: neutrophils treated with EVs-depleted RPMI-1640 medium. ^*^P < 0.05, ^**^P < 0.01, ^***^P < 0.001.

To evaluate whether or not EVs are involved in PD-L1 induction on neutrophils by GC microenvironment, we treated neutrophils isolated from human peripheral blood with GC cell-derived EVs (BGC-EVs) for 12 h. PD-L1 expression on the membrane of neutrophils was remarkably upregulated after treatment with BGC-EVs (Figure 1A). The increased expression of PD-L1 gene was also observed in BGC-EV-treated neutrophils compared to controls (Figure 1B). We further treated neutrophils with human gastric tumor tissue-derived EVs (TTEVs) or non-tumor tissue-derived EVs (NTEVs). Consistent with the above results, TTEVs significantly upregulated PD-L1 expression on neutrophils compared to NTEVs (Figure 1C). Real-time quantitative PCR analyses showed increased expression of PD-L1 gene in TTEV-treated neutrophils compared to NTEV-treated neutrophils (Figure 1D). These findings together imply that GC-derived EVs contribute to the increased expression of PD-L1 on neutrophils.

### Gastric Cancer-Derived Extracellular Vesicles Induce Programmed Death-Ligand 1 Expression on Neutrophils *via* Janus Kinase–Signal Transducer Activator of Transcription 3 Pathway

Emerging studies suggest that STAT3 activation induces the expression of PD-L1 in various cancers. The previous studies from others (21, 24) and our group (18, 19) have shown that STAT3 pathway is predominantly responsible for the activation of neutrophils by tumor signals. First, we demonstrated that JAK–STAT3 pathway was involved in GC-CM-induced PD-L1 expression on neutrophils (Figure S2). Next, we wanted to know whether STAT3 signaling pathway is involved in PD-L1 induction on neutrophils under the conditions of EVs.

**Figure 2:**
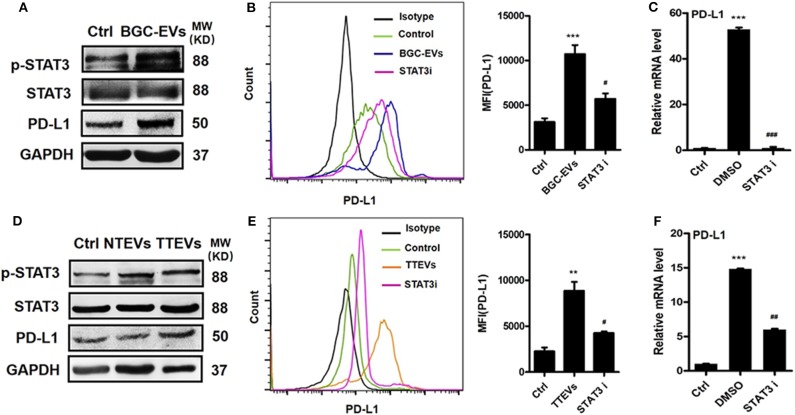
Gastric cancer (GC)-derived extracellular vesicles (EVs) induce programmed death-ligand 1 (PD-L1) expression on neutrophils *via* Janus kinase (JAK)–signal transducer and activator of transcription (STAT)3 pathway. **(A)** The expression of STAT3 and p-STAT3 in neutrophils treated with BGC-EVs for 12 h was determined by Western blot. Protein and gene levels of PD-L1 on neutrophils exposed to BGC-EVs in the presence or absence of WP1066 were determined by flow cytometry **(B)** and qRT-PCR **(C)**. **(D)** The expression of STAT3 and p-STAT3 in neutrophils treated with non-tumor tissue-derived EVs (NTEVs) and tumor tissue-derived EVs (TTEVs) for 12 h was determined by Western blot. Flow cytometric **(E)** and qRT-PCR analyses **(F)** of PD-L1 expression in neutrophils exposed to NTEVs and TTEVs with or without WP1066. Ctrl, neutrophils treated with EV-depleted RPMI-1640 medium. ^**^P < 0.01, ^***^P < 0.001; ^#^*P* < 0.05, ^##^*P* < 0.01, ^###^*P* < 0.001.

Neutrophils were exposed to GC-EVs and the activation of STAT3 signaling pathway was analyzed. We found that BGC-EV treatment increased the expression of p-STAT3 in neutrophils (Figure 2A). Meanwhile, TTEV-treated neutrophils showed more p-STAT3 expression than NTEV-treated neutrophils (Figure 2D). We pretreated neutrophils with STAT3 inhibitor and then exposed them to BGC-EVs or TTEVs. The results showed that blocking the signal transduction of JAK with inhibitor WP1066 effectively suppressed PD-L1 expression on BGC-EV- or TTEV-treated neutrophils (Figures 2B,E). The expression of PD-L1 gene was also downregulated in neutrophils pretreated with the inhibitor (Figures 2C,F). These data imply that JAK–STAT3 signaling pathway is involved in PD-L1 induction on neutrophils by GC-derived EVs.

### Neutrophils Activated by Gastric Cancer-Derived Extracellular Vesicles Suppress T Cell Immunity Through Programmed Death-Ligand 1

**Figure 3:**
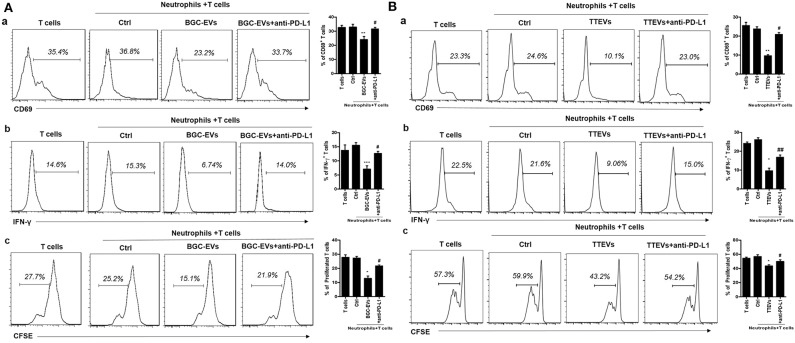
Neutrophils activated by gastric cancer (GC)-derived extracellular vesicles (EVs) suppress T cell immunity through programmed death-ligand 1 (PD-L1). Human peripheral CD3^+^ T cells were cocultured with **(A)** BGC-EV- or **(B)** tumor tissue-derived EV (TTEV)-treated neutrophils in the presence or absence of PD-L1 antibody. The expression of activation marker (CD69) (a), production of interferon (IFN)-γ (b), and proliferation (c) of T cells were determined by flow cytometry (*n* = 3). ^*^P < 0.05, ^**^P < 0.01, ^***^P < 0.001; ^#^*P* < 0.05, ^##^*P* < 0.01.

We next studied the biological roles of PD-L1 induction on neutrophils by GC microenvironment. Purified human peripheral CD3^+^ T cells were cocultured with neutrophils that had been treated with GC-CM. Interestingly, GC-CM-treated neutrophils significantly suppressed T cell activity (Figure S3). We further cocultured BGC-EV- and TTEV-treated neutrophils with T cells and found that BGC-EV- or TTEV-treated neutrophils significantly downregulated CD69 expression in T cells (Figures 3Aa,Ba). In addition, BGC-EV- or TTEV-treated neutrophils suppressed IFN-γ production and T cell proliferation (Figures 3Ab,c; Figures 3Bb,c). To determine the role of PD-L1 in the suppression of T cell immunity, we added neutralizing antibodies against PD-L1 in T cells/BGC-EV- or TTEV-treated neutrophils coculture system. Antibody blockade of PD-L1 efficiently attenuated the observed T cell suppression by BGC-EV- or TTEV-treated neutrophils. Collectively, these results indicate that neutrophils activated by GC-derived EVs are at least partially responsible for the induction of PD-L1 expression and immunosuppressive function in neutrophils.

### Gastric Cancer-Derived Extracellular Vesicles Induce Programmed Death-Ligand 1 Expression and Immunosuppressive Function in Neutrophils Through High-Mobility Group Box 1

**Figure 4:**
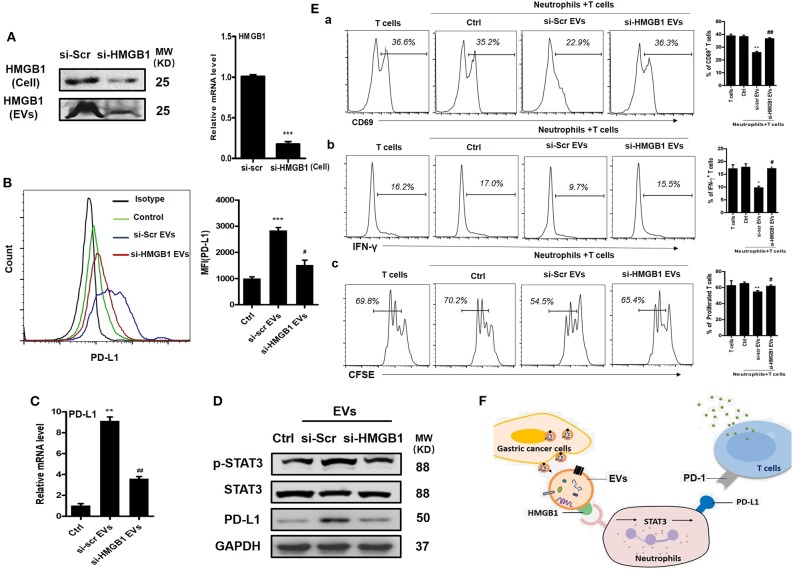
Gastric cancer (GC)-derived extracellular vesicles (EVs) induce programmed death-ligand 1 (PD-L1) expression and immunosuppressive function in neutrophils through high-mobility group box 1 (HMGB1). **(A)** The expression of HMGB1 in scramble and HMGB1 siRNA-transfected BGC cells and BGC-EVs was determined by Western blot and qRT-PCR. Protein and gene levels of PD-L1 on neutrophils exposed to si-scr or si-HMGB1 EVs for 12 h were determined by flow cytometry **(B)** and qRT-PCR **(C)**. **(D)** The expression of signal transducer and activator of transcription (STAT)3 and p-STAT3 in neutrophils treated with si-scr or si-HMGB1 EVs for 12 h was determined by Western blot. **(E)** Human peripheral CD3^+^ T cells were cocultured with si-scr or si-HMGB1 EV-treated neutrophils in the presence or absence of PD-L1 antibody. The expression of activation marker (CD69) (a), production of interferon (IFN)-γ (b), and proliferation (c) of T cells were determined by flow cytometry (*n* = 3). **(F)** Proposed model for GC-EV-induced PD-L1 expression on neutrophils and suppression of T-cell immunity in GC. Ctrl, neutrophils treated with EV-depleted RPMI-1640 medium. ^*^P < 0.05, ^**^P < 0.01, ***P < 0.001; ^#^*P* < 0.05, ^##^*P* < 0.01.

EVs can induce signaling pathway activation in recipient cells by delivering bioactive molecules. We previously performed a proteomic analysis for EVs from GC cells and identified several proteins of interest including HMGB1, which has shown important roles in tumor immune evasion and neutrophil polarization (18, 25, 26). To investigate whether HMGB1 is critical for GC-EV-induced PD-L1 expression on neutrophils, we silenced HMGB1 in GC cells by specific siRNA (Figure 4A). We then treated neutrophils with si-HMGB1 EVs to determine the role of si-HMGB1 EVs in PD-L1 expression on neutrophils. Compared with EVs from scramble siRNA-treated GC cells (si-scr EVs), si-HMGB1 EVs showed attenuated effect to induce PD-L1 expression on neutrophils both at protein and gene levels (Figures 4B–D). We also found that EVs from HMGB1-silenced cells could not activate STAT3 as si-scr EVs (Figure 4D). To further explore whether si-HMGB1 EV-treated neutrophils have the ability to suppress T cell immunity, we collected si-scr EV- and si-HMGB1 EV-treated neutrophils and cocultured with CD3^+^ T cells, respectively. Compared with si-scr EV-treated neutrophils, the suppressive effects of si-HMGB1 EV-treated neutrophils on activation of T cells and the proliferation and IFN-γ production of T cells were attenuated (Figure 4E). These findings suggest that EV-delivered HMGB1 is involved in the induction of immunosuppressive phenotype in neutrophils through STAT3/PD-L1 pathway in GC.

## Discussion

In this study, we provided the first evidence that GC cell-derived EVs induced PD-L1 expression on neutrophils. GC-EVs transported HMGB1 to activate STAT3 pathway and upregulate PD-L1 gene expression in neutrophils. We also provided the first demonstration that GC-EVs induced immunosuppressive neutrophils to inhibit T cell immunity through PD-L1/PD-1 interaction and that EV-delivered HMGB1 was responsible for the induction of immunosuppressive neutrophils. Our findings suggest an important role of EVs in establishing an immunosuppressive microenvironment, which provides new insight into the regulation of neutrophil biology and function in GC.

Immunosuppression is one of the hallmarks of cancer (27). The interaction between PD-L1 and PD-1 is one of the best-known mechanisms that are responsible for immunosuppression. Previous studies have shown that infiltrating neutrophils display a phenotype with high expression of PD-L1, and PD-L1^+^ neutrophils could effectively suppress the proliferation and activation of T cells (28). Consistent with the study, our results showed that the GC microenvironment induced a high expression of PD-L1 on neutrophils, indicating that neutrophils could be regulated by tumor signals to present an immunosuppressive phenotype.

PD-L1 expression is regulated by multiple factors in the context of infection, inflammation, and cancer. The regulation of PD-L1 expression in cancer is complex. In addition to inflammatory factors such as IL-10 and GM-CSF (21), other factors such as tumor cell-released autophagosomes (TRAPs) (29), long non-coding RNA HOXA transcript at the distal tip (HOTTIP) (30), and IL-6 from cancer-associated fibroblasts (CAFs) (31) have also been reported to regulate PD-L1 expression. Recently, EVs have been identified as an important factor to induce PD-L1 expression and impair anti-tumor immune. Tumor cell-derived EVs have been reported to induce high expression of PD-L1 *via* STAT3 pathway in macrophages (32) and monocytes (9), representing an immunosuppressive M2 phenotype. Here, we presented the first report that GC-EVs also could induce immunosuppressive neutrophils with increased expression of PD-L1 *via* activation of STAT3.

The molecular mechanisms for EV-mediated upregulation of PD-L1 seem to be complicated. EVs directly carry PD-L1 mRNA to enhance its transcription in melanoma and non-small-cell lung cancer (NSCLC) cells (33). In addition, exosomal non-coding RNAs including miR-23a-3p (34) and hY4 (9) upregulate PD-L1 expression in macrophages and monocytes. These molecules regulate PD-L1 gene expression to change PD-L1 protein levels in tumor cells or immune cells. However, metastatic melanoma (35), breast cancer (36), and head and neck squamous cell carcinoma (HNSCC) cells (37) release EVs that carry PD-L1 protein on their surface, which directly suppresses T cell killing of tumor cells and promote tumor growth. We reported that GC-EVs upregulated the transcription of PD-L1 by activating STAT3 since the inhibition of STAT3 activation decreases PD-L1 expression at both gene and protein level. Whether other forms of PD-L1 (mRNA, protein, or regulatory miRNAs) are contained in GC-EVs deserves further investigation.

HMGB1 is a critical immunosuppressive molecule in tumor microenvironment (38). In hepatocellular carcinoma (HCC), exosomal HMGB1 activates B cells with suppressive activity against CD8^+^ T cells and promotes TIM-1^+^ Breg cell expansion *via* the Toll-like receptor (TLR)2/4 and mitogen-activated protein kinase (MAPK) signaling pathways (25). We previously reported that GC-EV-transported HMGB1 interacts with TLR4 to activate neutrophils to promote GC cell migration (18). In this study, we revealed that GC-derived EVs transported HMGB1 to activate STAT3 pathway and induce PD-L1 expression on neutrophils, subsequently inhibiting the proliferation, activation, and function of T cells. These findings indicate that HMGB1, mainly in an extracellular vesical form, is an important regulator for PD-L1 expression and immune suppression. We also found that PD-L1 upregulation was not totally inhibited by blocking the HMGB1 pathway, suggesting that there may exist other suppressive pathways mediated by tumor-derived EVs. Fleming et al. (39) reported that EVs from melanoma cells transported heat-shock protein 86 (HSP-86) to upregulate PD-L1 expression on immature myeloid cells, leading to suppression of T cell activation. In addition, dendritic cells could uptake arginase-1 (ARG1)-containing EVs and inhibit antigen-specific T cell proliferation (40). These data suggest that the mechanisms for tumor-derived EVs in immune suppression are diverse. Specific antibody blockade study showed that GC-EV-elicited neutrophils suppressed the proliferation, activation, and function of T cells in a PD-L1-dependent manner. However, it could not be excluded that other factors from GC-EV-elicited neutrophils are also involved in this process.

In summary, based on our data, we proposed an *in vitro* model describing the role of GC-EVs in mediating immunosuppression through the regulation of neutrophils in GC (Figure 4F). EVs that carry HMGB1 are actively secreted into the GC microenvironment. The exosomal HMGB1 induces PD-L1 expression on neutrophils *via* activation of STAT3 signaling pathway. The PD-L1^+^ neutrophils exert a pro-tumor effect by suppressing T cell function through PD-L1/PD-1 interaction. In the future, a potential strategy that interferes with the EV-derived HMGB1–STAT3–PD-L1 pathway may be developed to treat GC.

## Data Availability Statement

The datasets generated for this study are available on request to the corresponding author.

## Ethics Statement

The studies involving human participants were reviewed and approved by the Ethics Committee Jiangsu University. The patients/participants provided their written informed consent to participate in this study.

## Author Contributions

YS and XZ conceived the idea and designed the study. YS, JZ, and ZM performed the experiments and collected the data. YS, JZ, HS, RJ, HQ, and WX analyzed and interpreted the data. All of the authors took part in the writing of the paper.

## Conflict of Interest

The authors declare that the research was conducted in the absence of any commercial or financial relationships that could be construed as a potential conflict of interest.

## Supplementary Material

Figure S1 Human GC microenvironment induces PD-L1 expression on neutrophils. (A and B) Protein and gene levels of PD-L1 on neutrophils exposed to BGC-CM for 12 hours were determined by flow cytometry. **(A)** and qRT-PCR **(B)**. (C and D) Flow cytometric **(C)** and qRT-PCR analyses **(D)** of protein and gene levels of PD-L1 in neutrophils exposed to NTCS and TTCS for 12 hours. MFI: mean fluorescence intensity. Ctrl: neutrophils treated with exosome-depleted RPMI-1640 medium. ^*^*P* < 0.05, ^**^*P* < 0.01, ^***^*P* < 0.001.

Figure S2 Human GC microenvironment induces PD-L1 expression on neutrophils via JAK-STAT3 pathway. **(A)** The expression of STAT3 and p-STAT3 in neutrophils treated with BGC-CM for 12 hours was determined by western blot. (B and C) Protein and gene levels of PD-L1 on neutrophils pre-treated with or without JAK-STAT3 inhibitor WP1066 followed by exposure to BGC-CM were determined by flow cytometry **(B)** and qRT-PCR **(C)**. **(D)** The expression of STAT3 and p-STAT3 in neutrophils with NTCS and TTCS for 12 hours was determined by Western blot. (E and F) Flow cytometric **(E)** and qRT-PCR analyses **(F)** of PD-L1 expression in neutrophils exposed to NTCS and TTCS with or without WP1066. Ctrl: neutrophils treated with exosome-depleted RPMI-1640 medium. ^*^*P* < 0.05, ^**^*P* < 0.01, ^***^*P* < 0.001. ^#^*P* < 0.05, ^*##*^*P* < 0.01, ^*###*^*P* < 0.001.

Figure S3 Neutrophils activated by GC microenvironment suppress T cell immunity through PD-L1. (A and B) Human peripheral CD3+ T cells were co-cultured with **(A)** BGC-CM or **(B)** TTCS treated neutrophils in the presence or absence of PD-L1 antibody. (a, b, c) The expression of activation marker (CD69), production of IFN-γ, and proliferation of T cells were determined by flow cytometry (*n*=3). CFSE, carboxyfluorescein succinimidyl ester. ^*^*P* < 0.05, ^**^*P* < 0.01, ^***^*P* < 0.001. ^#^*P* < 0.05, ^*##*^*P* < 0.01, ^*###*^*P* < 0.001.
