# Supplementary figures and images for: Extracellular Vesicles From Gastric Cancer Cells Induce PD-L1 Expression on Neutrophils to Suppress T-Cell Immunity
Source: Front Oncol. 2020 May 13;10:629. doi: 10.3389/fonc.2020.00629 (PMC7237746; doi:10.3389/fonc.2020.00629)

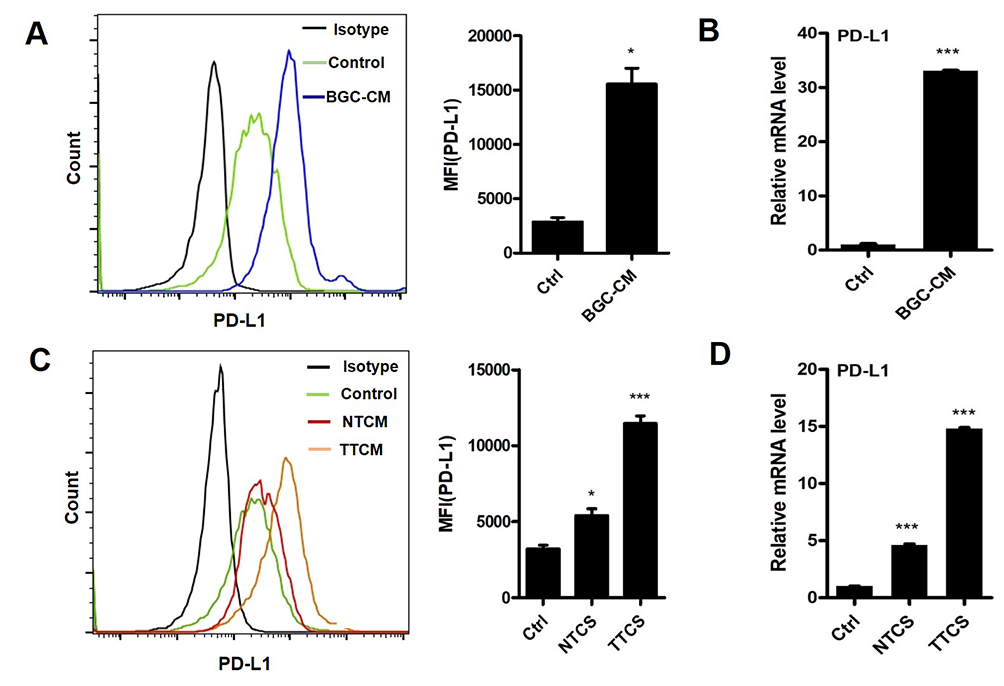

Supplement: Figure S1 — Human GC microenvironment induces PD-L1 expression on neutrophils. (A and B) Protein and gene levels of PD-L1 on neutrophils exposed to BGC-CM for 12 hours were determined by flow cytometry. (A) and qRT-PCR (B). (C and D) Flow cytometric (C) and qRT-PCR analyses (D) of protein and gene levels of PD-L1 in neutrophils exposed to NTCS and TTCS for 12 hours. MFI: mean fluorescence intensity. Ctrl: neutrophils treated with exosome-depleted RPMI-1640 medium. *P < 0.05, **P < 0.01, ***P < 0.001. [file Image_1.TIF]

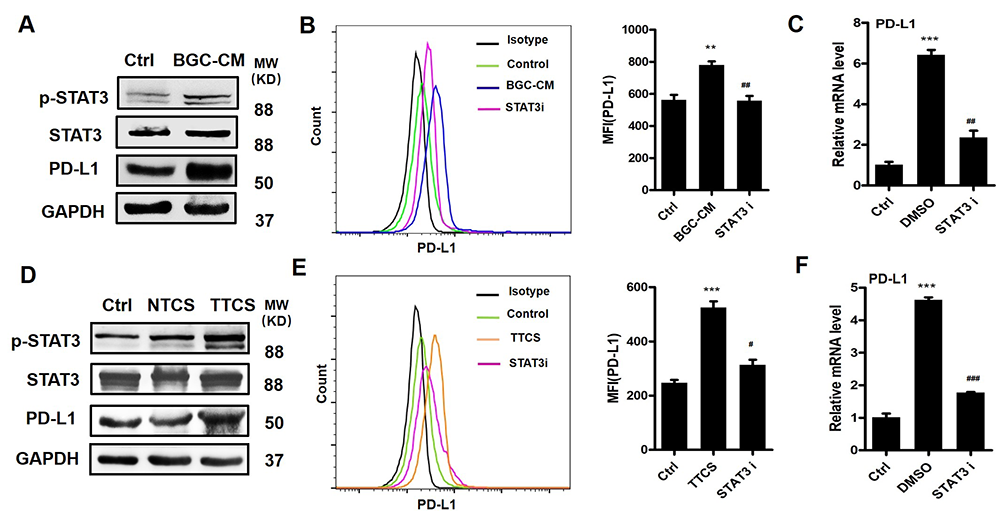

Supplement: Figure S2 — Human GC microenvironment induces PD-L1 expression on neutrophils via JAK-STAT3 pathway. (A) The expression of STAT3 and p-STAT3 in neutrophils treated with BGC-CM for 12 hours was determined by western blot. (B and C) Protein and gene levels of PD-L1 on neutrophils pre-treated with or without JAK-STAT3 inhibitor WP1066 followed by exposure to BGC-CM were determined by flow cytometry (B) and qRT-PCR (C). (D) The expression of STAT3 and p-STAT3 in neutrophils with NTCS and TTCS for 12 hours was determined by Western blot. (E and F) Flow cytometric (E) and qRT-PCR analyses (F) of PD-L1 expression in neutrophils exposed to NTCS and TTCS with or without WP1066. Ctrl: neutrophils treated with exosome-depleted RPMI-1640 medium. *P < 0.05, **P < 0.01, ***P < 0.001. #P < 0.05, ##P < 0.01, ###P < 0.001. [file Image_2.TIF]

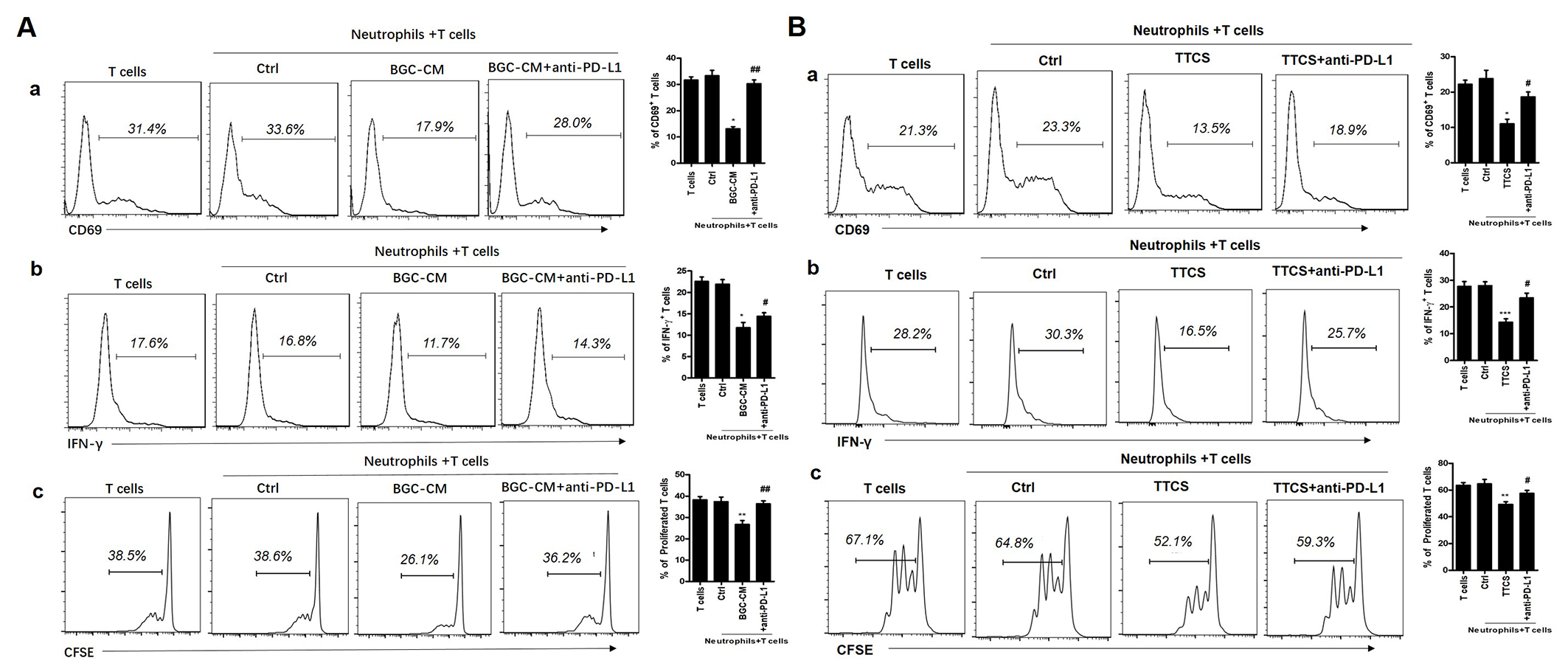

Supplement: Figure S3 — Neutrophils activated by GC microenvironment suppress T cell immunity through PD-L1. (A and B) Human peripheral CD3+ T cells were co-cultured with (A) BGC-CM or (B) TTCS treated neutrophils in the presence or absence of PD-L1 antibody. (a, b, c) The expression of activation marker (CD69), production of IFN-γ, and proliferation of T cells were determined by flow cytometry (n=3). CFSE, carboxyfluorescein succinimidyl ester. *P < 0.05, **P < 0.01, ***P < 0.001. #P < 0.05, ##P < 0.01, ###P < 0.001. [file Image_3.TIF]
